# Gut Microbe Fermentation of *Moringa oleifera* Leaf Extract Increases Measurable Polyphenols and Improves Barrier Function in a Cell Culture Model

**DOI:** 10.1002/mbo3.70068

**Published:** 2025-11-24

**Authors:** Mary E. Kable, David H. Storms, Zeynep Alkan, Maneesha Muriki, Dana DeVries, Carrie Waterman, Danielle G. Lemay

**Affiliations:** ^1^ USDA, Agricultural Research Service, Western Human Nutrition Research Center, Diet Microbiome and Immunity Unit Davis California USA; ^2^ Department of Nutrition University of California, Davis Davis California USA; ^3^ University of California, Davis, Global Disease Biology Program Davis California USA; ^4^ University of California, Davis, Institute for Global Nutrition Davis California USA

## Abstract

*Moringa oleifera* is associated with several nutritional and therapeutic benefits. However, there is limited research on how much these health benefits are mediated directly by the plant or through fermentation with intestinal microbes. We examined the interaction between *M. oleifera* aqueous leaf extract and three common gut microbes whose abundance was significantly altered in previous intervention studies. Growth curves of *Escherichia coli*, *Bifidobacterium longum*, and *Bacteroides thetaiotaomicron* were examined in the presence of increasing concentrations of *M. oleifera* leaf extract in YCFA media with and without carbohydrates and short chain fatty acids (SCFAs). Anthrone and Fast Blue BB assays were conducted on spent media to measure carbohydrate and phenolic content, respectively. Sterile fermentation supernatants were applied to an in vitro gut barrier model consisting of differentiated Caco‐2 monolayers on permeable cell culture inserts and the transepithelial resistance (TEER) was measured. Growth curve analysis demonstrated that the three bacterial isolates tested could grow in the presence of *M. oleifera*. However, *B. longum* had a greater increase in total growth, consumed more soluble carbohydrates, and produced more soluble polyphenols using *M. oleifera* leaf extract as a sole carbohydrate source than the other two microbes. Additionally, *B. longum* fermentation of both glucose and *M. oleifera* increased TEER in Caco‐2 monolayers significantly more than *E. coli* fermentation of both carbohydrate sources (*p* = 0.0007). These results suggest a potential mechanism through which consumption of *M. oleifera* may promote the growth of probiotic organisms within the human gut to improve gut barrier integrity.

## Graphical abstract

Three gut bacteria isolates were tested in vitro for their ability to grow and produce detectable polyphenols during fermentation of *M. oleifera* aqueous leaf extract. *B. longum* had the highest increase of soluble polyphenols during fermentation and *B. longum*‐fermented *M. oleifera* significantly increased TEER readings in a Caco‐2 monolayer.

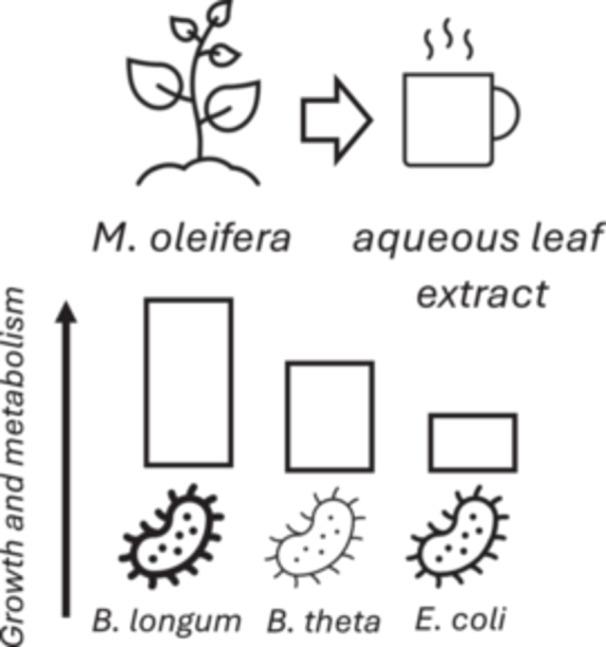

## 1 Introduction

*Moringa oleifera* is an edible nutrient‐dense medicinal plant native to India and Pakistan (Patil et al. 2022). It is a member of the Moringaceae family in the order Brassicales, which includes common vegetables like broccoli, cabbage, and mustard greens. Extracts from the roots, flowers, bark, seeds, and leaves have been shown to possess antioxidant, anti‐inflammatory, and antimicrobial properties (Dzotam et al. 2016; Pareek et al. 2023). *M. oleifera* leaves have been investigated as a means to help treat hyperglycemia and dyslipidemia (Mbikay 2012), malnutrition (Kasolo et al. 2010), and cancer (Al‐Asmari et al. 2015). These proposed nutritional and medicinal benefits are thought to be mediated largely by the abundant and diverse nutrient and phytochemical content of the plant including its unique glucosinolates (Rocchetti et al. 2019; Lopez‐Rodriguez et al. 2020).

Extracts of dried *M. oleifera* leaf have been reported to contain all major phytochemical classes tested: alkaloids, polyphenols, flavonoids, glucosinolates, anthraquinones, coumarins, tannins, triterpenes, sterols, and saponins (Rocchetti et al. 2019; Lopez‐Rodriguez et al. 2020). However, hydroxybenzoic acid, protocatechuic acid, and quercetin glycosides are the dominant polyphenols found (Mbikay 2012; Rocchetti et al. 2019). While methanol extraction is most efficient (Rocchetti et al. 2019), water extraction can result in polyphenol yields ranging from 1.1% to 4.5% of total dry matter (Dou et al. 2019; Waterman et al. 2014; Chumark et al. 2008).

In addition to being a rich source of potentially bioactive phytochemicals, *M. oleifera* also contains abundant soluble fiber (Peñalver et al. 2022; Melesse et al. 2013). Consumption of soluble dietary fiber promotes increases in the relative abundance of probiotic bacteria, including bifidobacteria. It is likely that the dietary fiber component of *M. oleifera* may play a role in various metabolic processes to promote health. *Bifidobacterium* spp. have been observed to increase in response to *M. oleifera* supplementation in the diets of both animals and humans (Gao et al. 2017; Bisanz et al. 2015; Khalid et al. 2022). The abundance of *Bacteroides* has been reported to be affected both positively (ex vivo fermentation of human stool, and rabbit and chicken intervention diets) and also negatively in a dairy cow intervention diet (Khalid et al. 2022; Soundararajan et al. 2023; Jiang et al. 2023; Sun et al. 2017; Li et al. 2021) with *M. oleifera* supplementation. On the other hand, *E. coli*, *Streptococcus* spp. and *Staphylococcus* spp. are commonly decreased by *M. oleifera* both during in vitro testing and feeding studies (El‐Badawi et al. 2018; Viera et al. 2010; Al_husnan and Alkahtani 2016; Abu Hafsa et al. 2020). *Bacteroides* spp. are one of the most dominant bacterial taxa within the human gut and the *Bifidobacterium* genus is one of the most widely studied, and potentially beneficial. All three bacterial genera are present in the human gastrointestinal tract and therefore have the potential to interact with *M. oleifera* to influence human health.

Therefore, to understand how *M. oleifera* might interact with gut microbes abundant in the colon of U.S. adults, the behavior of *Escherichia coli*, *Bifidobacterium longum*, and *Bacteroides thetaiotaomicron* were examined in this study study.

## 2 Methods

### 2.1 Bacterial Isolates and Culture Conditions

Bacterial isolates, *Bacteroides thetaiotaomicron* ATCC 29148, *Escherichia coli* ATCC 25922, and *Bifidobacterium longum* subsp. *longum* ATCC 15707, were grown in yeast casitone fatty acid (YCFA) media (Duncan et al. 2002)]. Complete media consisted of yeast extract (2.5 g/L), casitone (10 g/L), sodium chloride (NaCl, 0.9 g/L), ammonium sulfate ((NH_4_)_2_SO_4_, 0.9 g/L), magnesium sulfate heptahydrate (MgSO_4_ • 7H_2_O, 0.09 g/L), calcium chloride (CaCl_2_, 0.09 g/L), potassium dihydrogen phosphate (KH_2_PO_4_, 0.45 g/L), sodium bicarbonate (NaHCO_3_, 40 g/L), cysteine (1 g/L), resazurin (1 mg/L), hemin (10 mg/L), biotin (10 µg/L), cobalamin B12 (1 µg/L), p‐aminobenzoic acid (30 µg/L), folic acid (50 µg/L), pyridoxamine dihydrochloride (150 µg/L), thiamine (50 µg/L), riboflavin (50 µg/L), d‐glucose (2 g/L), maltose (2 g/L), d‐cellobiose (2 g/L), glacial acetic acid (1.89 g/L), propionic acid (0.68 g/L), isobutyric acid (0.09 g/L), isovaleric acid (0.11 g/L), and valeric acid (0.11 g/L). Before use, media was adjusted to pH 7, filtered through a 0.2 µm PES filter (Corning), and equilibrated for 3 d under anaerobic conditions (2‐3% H_2_, 10% CO_2_, balance N_2_).

### 2.2 *M. oleifera* Leaf Extract Preparation

Dried *M. oleifera* leaves, grown in Fresno and Tulare counties, CA and supplied by UC ANR, were powdered in a kitchen blender (Oester) at maximum speed for 2 min under sterile conditions. Leaf powder was extracted at a 1:10 (w:v) ratio in sterile water for 48 h at 30°C protected from light. Solids were removed by pelleting three times at 5000 × g for 10 min at 4°C. The resultant supernatant was dried using a speed vacuum concentrator (Genevac EZ‐2 Plus, SP Scientific, Ipswich, UK) at room temperature and stored at 4°C pending use. All subsequent experiments used the same original powdered lot of *M. oleifera* leaves.

### 2.3 Bacterial Growth Assays

The effects of *M. oleifera* leaf extract on bacterial growth kinetics were assessed using three variants of YCFA media: complete (described above), lacking carbohydrates, and lacking both carbohydrates and SCFAs. Dried *M. oleifera* leaf extract was reconstituted in each YCFA media variant, filtered through a 0.2 µm PES filter, and serially diluted with the same media in a twofold dilution series to the following final concentrations: 0, 0.78, 1.56, 3.13 6.25, 12.5, 25, and 50 mg/mL. Bacterial isolates grown overnight in complete media were washed and transferred to the YCFA media type of interest (complete, lacking carbohydrates and/or SCFAs) at 0.01 OD_600_.

Growth kinetics of each bacterial isolate were measured in the presence of *M. oleifera* leaf extract under anaerobic conditions. Absorbance (600 nm) was monitored in 200 µL culture volumes using a 96‐well plate (Corning) at 10 min intervals for 42 h at 37°C using a SPECTROstar Nano (BMG Labtech). The plate was loosely covered, and outer wells filled with sterile buffer to limit evaporation. Three independent experiments with duplicate wells were conducted per growth condition. Maximal growth was defined as maximum minus minimum optical density measured during 42 h of monitored growth. Maximal growth rate was determined as the maximum slope of optical density versus time during the exponential growth phase of the isolates, usually within the first 10 h.

### 2.4 Carbohydrate and Phenolic Assays of *M. oleifera* Fermentations

Utilization of carbohydrates and changes to phenolic content by bacterial isolate fermentation of *M. oleifera* leaf extracts were assessed in the terminal supernatants of the growth experiments described above. Supernatants were prepared by pooling duplicate wells, centrifuging at 10,000 × g for 10 min at 4°C and assayed in triplicate. Sham‐inoculated sterility controls were also assayed to discriminate between incubation and fermentation effects. Anthrone‐sulfuric acid assay was conducted as described previously [6]. Briefly, 50 µL glucose standard (0 to 600 mg/L) or diluted supernatant was incubated in a polypropylene 96‐well plate (Corning) with 150 µL freshly prepared 2 mg/mL anthrone in 98% sulfuric acid for 10 min at 4°C, 10 min at 100°C, and 20 min at 25°C before measuring absorbance at 620 nm (Synergy2, BioTek). Phenolic content was likewise determined with phenolic assay Fast Blue BB (Lester et al. 2012), which was conducted by incubating 100 µL gallic acid standard (0–5 mM) or diluted sample with 40 µL of sterile aqueous suspension of 0.1% Fast Blue BB for 5 min at 25°C before developing for 90 min with 60 µL 1.67% NaOH and measuring absorbance at 450 nm.

### 2.5 *M. oleifera* Leaf Extract Fermentations for Application to an In Vitro Gut Barrier Model

Fifty mg/mL of dried *M. oleifera* aqueous leaf extract was prepared in YCFA medium lacking SCFAs and carbohydrates (YCFA‐NCS) as described above and then anaerobically fermented in 2 mL culture volume for 48 h with each bacterial isolate. The same medium without *M. oleifera* leaf extract was supplemented with 6 g/L glucose and fermented in parallel to act as a negative control. Bacterial isolate preparation for inoculation was performed identically as for the bacterial kinetic growth assays. Post fermentation, supernatants were neutralized to pH 7 with 1 M NaOH, balanced with 1 M NaCl to produce equivalent salt concentrations, and filtered through 0.2 μm PES filters (Corning) before freezing. Each fermentation was performed independently in triplicate.

### 2.6 In Vitro Gut Barrier Model

Caco‐2 human epithelial colorectal adenocarcinoma cells (HTB‐37, ATCC) were grown in Dulbecco's Modified Eagle Medium (DMEM, Corning) containing 4.5 g/L glucose and sodium pyruvate supplemented with 10% FBS (Invitrogen), 1% GlutaMAX (Gibco), 1% non‐essential amino acids (Sigma), and 1% antibiotic‐antimycotic (Gibco) at 37°C and under 5% CO_2_. One hundred thousand undifferentiated Caco‐2 cells (passages 19–25) were seeded per 12 mm Transwell cell culture insert with a 0.4 µm pore size (catalog # 3493, Corning) on the apical side, and allowed to reach confluence and differentiate for 14 days. Cells were maintained with 0.5 mL and 1.5 mL complete DMEM on apical and basolateral Transwell sides, respectively. Apical and basolateral media were changed three times per week, and on the day before experimental treatments. TEER of Caco‐2 layers was measured after Transwells were rinsed with Phenol Red‐free Hank's Balanced Salt Solution (HBSS) at pre‐ and 24 h posttreatment time points. TEER measurements were conducted with an EVOM2 Epithelial Volt/Ohm Meter (World Precision Instruments) by placing each Transwell insert inside a sterile ENDOHM‐12 chamber and using Phenol Red‐free HBSS as electrode buffer at room temperature. Pretreatment resistance of Caco‐2 layers was 250–350 ohms on day 14 after seeding into Transwell inserts. Two‐hundred and fifty micro liter of filter‐sterilized fermentation supernatants or control treatments in HBSS were applied to the apical sides of Transwells in duplicate, and duplicate treatments were spread across different 12‐well companion plates to account for any plate‐to‐plate variation. 2 h after the application of apical treatments, TNF‐α at a final concentration of 50 ng/mL was added to media in all basolateral compartments, except for the mock‐treated Transwells. Three sets of independent fermentations were tested in three independent gut barrier model experiments.

### 2.7 Statistical Analysis

Fermentations and in vitro gut barrier experiments were performed three independent times. Kinetic growth curves were fit to a five‐parameter logistic function. Absorbance values for total bacterial growth and maximal growth rate at varying concentrations of *M. oleifera* leaf extract were transformed to minimize the skew of residuals and analyzed using a repeated measures ANOVA. This was performed by running ANOVA function from the R package stats v 4.1.1 on a linear mixed effects model, which was fit using lme function in R package nlme v 3.1‐152 with experiment as the random effect. Pairwise comparisons were performed with Dunnett's posttest, using the glht function in R package multcomp v 1.4–17, comparing each leaf extract concentration to 0 mg/mL extract. *P*‐values were adjusted for multiple comparisons using Bonferroni correction.

Comparisons of changes in maximal growth, and percent change in carbohydrate or polyphenol consumption among bacterial isolates were analyzed by two‐way ANOVA using the ANOVA function on a linear model, fit with the lm function from R package stats v 4.1.1. Pair‐wise comparisons were performed using estimated marginal means posttest and *p*‐values adjusted using the Tukey method for comparing a family of three estimates with emmeans function in the R package emmeans v 1.7.2

For the analysis of TEER data, bacterium and fermented nutrient (*M. oleifera* vs. glucose) interactions were determined with a two‐way ANOVA (R package stats v 4.0.3) using untransformed percent change in TEER (from pre‐ to 24 h posttreatment), followed by Tukey multiple comparisons of means. Mock and positive control treatments were excluded from ANOVA due to unbalanced design. Paired *t*‐tests were used to compare control treatments to fermentations (R package stats v 4.0.3).

## 3 Results

### 3.1 Effects of *M. oleifera* Leaf Extract on Bacterial Isolate Growth

**Figure 1:**
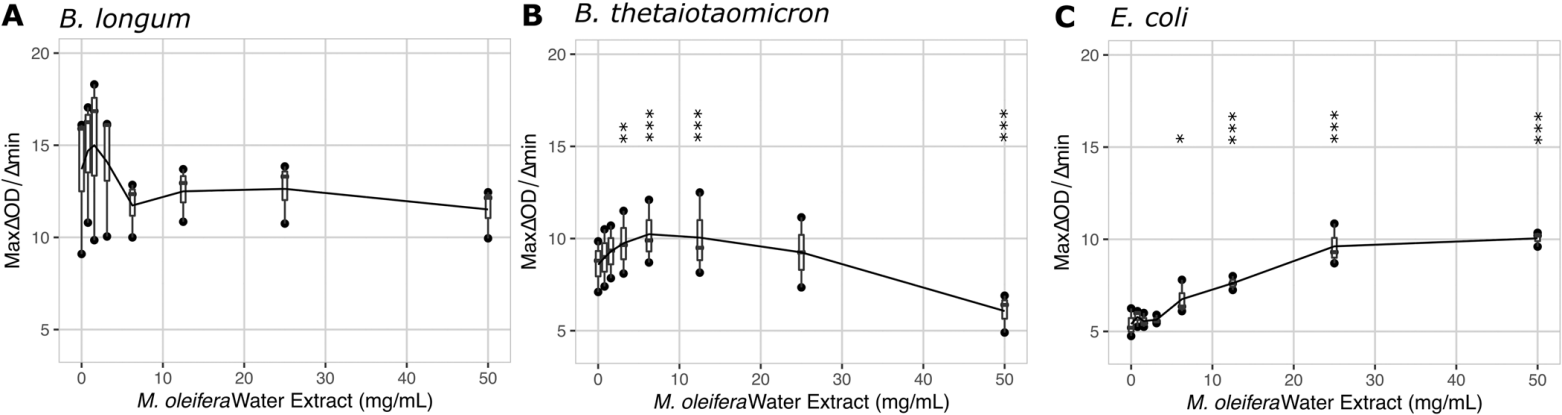
Growth rate of bacterial isolates exposed to *M. oleifera* leaf extract in complete YCFA medium. Maximal growth rate, shown on the y‐axis, was measured by rate of change in OD600 of bacterial isolates *B*. *longum* (A), *B. thetaiotamicron* (B), and *E. coli* (C) in complete YCFA media containing *M. oleifera* leaf extract concentrations shown on the *x*‐axis. *B. thetaiotamicron* showed inhibited growth rate at the highest extract dose. Results of repeated measures ANOVA (P shown on graph) with Dunnett's posttest using growth rate at 0 mg/mL as a reference to compare to growth rate at each extract concentration using untransformed data. Bonferroni correction was performed for multiple comparisons. The vertical lines on the graph show the standard error of the mean between the three replicates at each *M. oleifera* concentration. Statistical Significance: *p* < 0.05 *, *p* < 0.01 **, *p* < 0.001 ***.

Kinetic growth assays conducted for 42 h in complete YCFA medium spiked with twofold dilutions of *M. oleifera* leaf extract up to 50 mg/mL showed that leaf extract was not sufficient to inhibit maximal growth of *E. coli*, *B. longum*, or *B. thetaiotaomicron* cultured as isolates at these *M. oleifera* concentrations. Instead, total growth of each isolate was significantly increased at concentrations above 6.25 mg/mL *M. oleifera* leaf extract (Supporting Information: Figure S1). The one exception was *B. thetaiotamicron*, which had no significant change in total growth at 50 mg/mL *M. oleifera* leaf extract relative to 0 mg/mL (Supplemental Figure 1B). Maximal growth rate of *B. longum* was moderately affected (ANOVA *p* = 0.042), but no pairwise comparison between growth on 0 mg/mL *M. oleifera* and any other concentration were statistically significant (Figure 1A). On the other hand, maximal growth rate for *B. thetaiotamicron* was significantly increased at low concentrations of *M. oleifera* leaf extract (3.13–12.5 mg/mL) and significantly reduced at the highest *M. oleifera* leaf extract concentration (50 mg/mL) (Figure 1B). Surprisingly, the growth rate for *E. coli* was significantly increased at *M. oleifera* concentrations from 6.25 to 50 mg/mL (Figure 1C).

**Figure 2:**
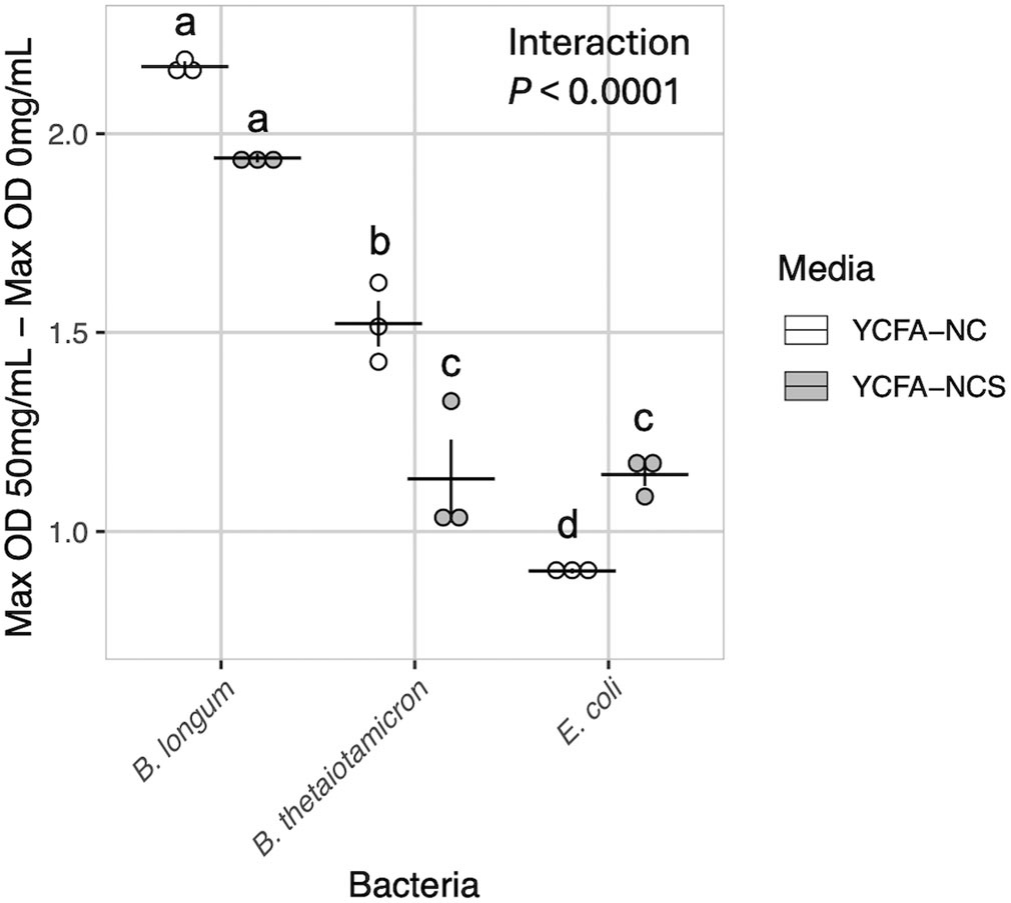
Effects of *M. oleifera* leaf extract concentration on maximal growth of bacterial isolates when reconstituted in media lacking carbohydrates. Difference between maximal growth of bacterial isolates measured by net A_600_ in either YCFA‐NCS (media lacking carbohydrates) or YCFA‐NC (media lacking carbohydrates and SCFAs), containing 50 and 0 mg/mL *M. oleifera* leaf extract after 42 h. Three independent experiments per bacterial isolate are shown. Two‐way ANOVA of Tukey's Ladder of Powers transformed values with estimated marginal means posttest and p‐values adjusted using the Tukey method for comparing a family of three estimates. Statistical significance: There was a significant difference in the growth of each bacterial strain (ANOVA, *P* = 5.387 × 10^−09^). There was a significant interaction between bacterial strain and media type (ANOVA, *P* = 4.440 × 10^−05^). However, there was no significant difference in growth by media alone (ANOVA, *p* = 0.2767). Growth outcomes not sharing a letter are statistically different from each other by posttest with *p*‐value < 0.05. Horizontal lines indicate mean values of each group. Vertical lines indicate standard error of the three replicate points within each group.

To understand whether each bacterial isolate could utilize *M. oleifera* leaf extract as a carbohydrate source, carbohydrates and SCFAs were omitted from media (YCFA‐NCS) used to reconstitute the *M. oleifera* leaf extract and kinetic growth assays were repeated. All bacterial isolates could use the M. *oleifera* leaf extract as a carbohydrate source, displaying significantly increased growth at all concentrations tested relative to 0 mg/mL *M. oleifera* leaf extract or media only (Supporting Information: Figure S2). Examination of maximum total growth in media with 50 mg/mL *M. oleifera* leaf extract lacking carbohydrates only (YCFA‐NC) or lacking both carbohydrates and SCFAs (YCFA‐NCS) showed that B. *longum* had significantly greater increase in maximal growth between 0 and 50 mg/mL *M. oleifera* leaf extract in both media types relative to the other two isolates (Figure 2). Further, both *B. longum* and *B. thetaiotamicron* showed significantly improved growth with 50 mg/mL *M. oleifera* in the presence of SCFAs (YCFA‐NC) relative to media lacking SCFAs (YCFA‐NCS). However, *E. coli* showed significantly reduced growth in the presence of SCFAs at the same concentration of *M. oleifera*.

### 3.2 Changes in Carbohydrate and Polyphenol Content During Bacterial Fermentation of *M. oleifera* Leaf Extract

**Figure 3:**
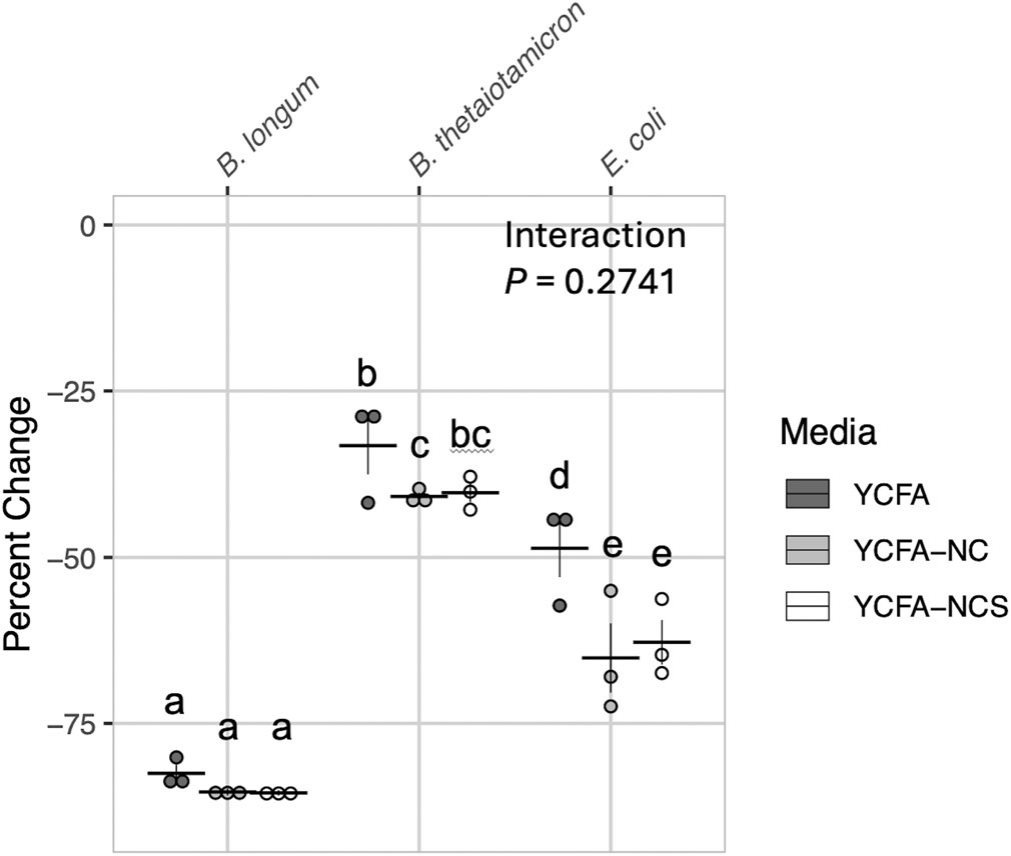
Carbohydrate use by bacterial isolates fermenting 50 mg/mL *M. oleifera* leaf extract for 42 h. Carbohydrate consumption by bacterial isolates was determined by assaying anthrone‐reactive substances in complete YCFA, YCFA lacking carbohydrates (YCFA‐NC), and YCFA lacking both SCFAs and carbohydrates (YCFA‐NCS), all containing 50 mg/mL *M. oleifera* leaf extract. Media incubated for 42 h but not inoculated with leaf extract was used to determine baseline carbohydrate content for evaluating these changes. All experiments were conducted independently in triplicate. Results of 2‐way ANOVA with pair‐wise comparison of estimated marginal means posttest of Tukey's Ladder of Powers transformed values. P‐values adjusted using the Tukey method for comparing a family of three estimates. Statistical significance: There was a significant difference in carbohydrate utilization of each bacterial strain (ANOVA, *P* = 2.853 × 10^−11^) and among media type (ANOVA, *P* = 0.003164). However, there was no significant interaction between media and bacterial strain for carbohydrate utilization (ANOVA, *P* = 0.2741). Groups not sharing a letter are statistically different from each other by posttest with *p*‐value < 0.05. Horizontal lines indicate mean values of each group. Vertical lines indicate standard error of the three replicate points within each group.

Residual carbohydrates were assayed after fermentation for 42 h of 50 mg/mL *M. oleifera* in each of the media types and compared to incubated but uninoculated media. The amount of carbohydrate utilized was significantly different for each bacterium (2‐way ANOVA of Tukey's Ladder of Powers transformed values, *P* = 2.853 × 10^−^^11^), and carbohydrate utilization was significantly different among media types (*p* = 0.003164). However, there was no significant interaction between media type and bacterial isolate. *B. longum* fermented the greatest proportion of carbohydrates at more than 75% in all media types (pair‐wise comparisons using estimated marginal means, emmeans package in R, *p* < 0.01 for both *B. thetaiotamicron* and *E. coli*) while *B. thetaiotaomicron* fermented the smallest proportion (Figure 3). Across all three bacterial isolates, complete YCFA media containing 6 g/L glucose, maltose, and cellobiose in addition to the water‐soluble carbohydrates in the *M. oleifera* leaf extract had significantly more residual carbohydrates remaining after fermentation than the other two media types (*p* = 0.01 for YCFA‐NCS; *p* = 0.005 for YCFA‐NC).

**Figure 4:**
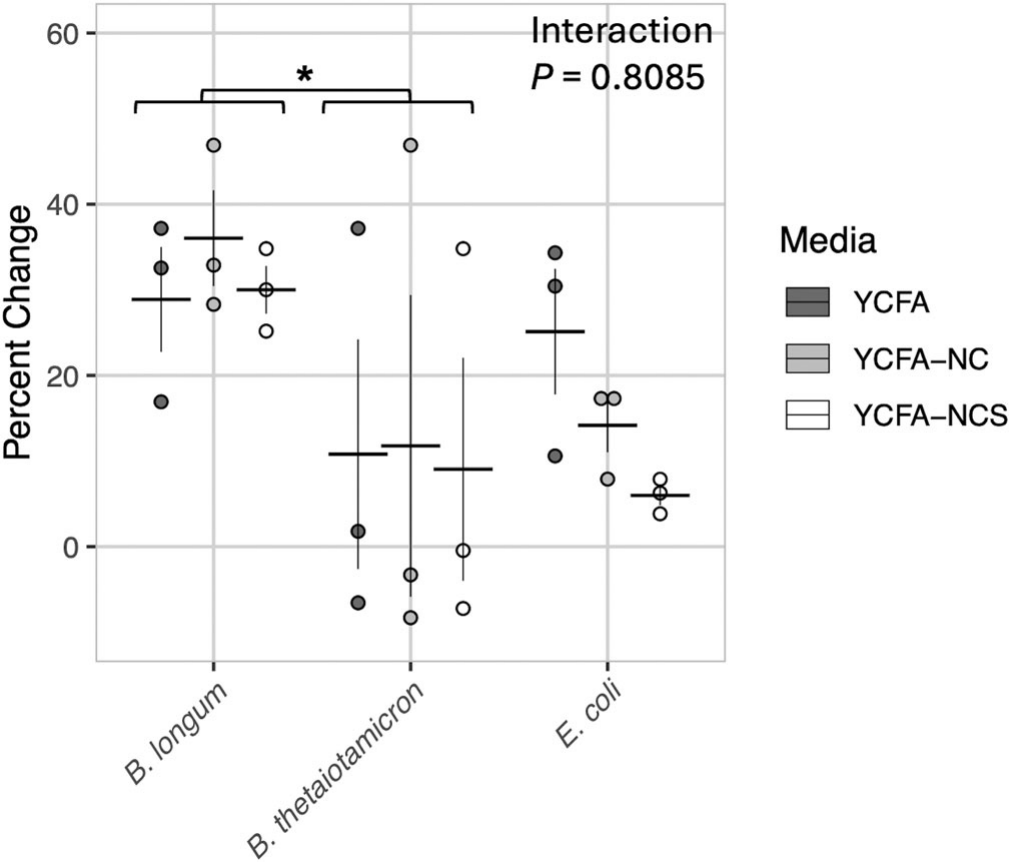
Changes to phenolic content by bacterial isolates in three YCFA media types. Total phenolic content was determined by Fast Blue BB assay after 42 h bacterial fermentation at 37°C of complete YCFA, YCFA‐NC, and YCFA‐NCS, all containing 50 mg/mL *M. oleifera* leaf extract. Uninoculated media with *M. oleifera* leaf extract was considered baseline for these changes. All experiments were conducted independently in triplicate. Results of 2‐way ANOVA with pair‐wise comparison of estimated marginal means posttest. *p*‐values adjusted using the Tukey method for comparing a family of three estimates. *p < 0.05.

Total phenolic content of fermented *M. oleifera* leaf extract at 50 mg/mL in each media variant was also measured using Fast Blue BB diazonium salt after 42 h of incubation with each bacterial isolate. *B. longum* showed a significant increase in total phenolic content during fermentation of *M. oleifera* leaf extract in all media types (Figure 4). There was a significant difference in total polyphenol content by bacterial isolate (*p* = 0.03296), but not media type (*p* = 0.65875) by two‐way ANOVA on untransformed values (Shapiro‐Wilk *p*‐value = 0.08). The phenolic content was highly variable after fermentation with *B. thetaiotaomicron*, increasing during some experimental replicates and decreasing in others, but overall was significantly lower than with *B. longum* (*p* = 0.0345). The polyphenol content of *M. oleifera* leaf extract fermentation with *E. coli* was not significantly different from the other bacterial isolates, polyphenol metabolism by this bacterium was seemingly affected by both the total carbohydrate and SCFA content of the media. However, this trend that appears visually was not supported statistically.

### 3.3 Effects of Glucose and *M. oleifera* Fermentations on Caco‐2 TEER

We used an in vitro gut barrier model to assess the effect of bacterial fermentations of *M. oleifera* relative to glucose on the integrity of Caco‐2 monolayers challenged with TNF‐ α. Fifty mg/mL dried *M. oleifera* leaf extract or 6 g/L glucose in YCFA‐NCS medium were fermented by *E. coli*, *B. thetaiotaomicron*, or *B. longum* in a 2 mL volume for 48 h. Both the pH and salt concentrations were adjusted to be equivalent between fermentation supernatants, and fermentations were sterile filtered before application to transwells.

**Figure 5:**
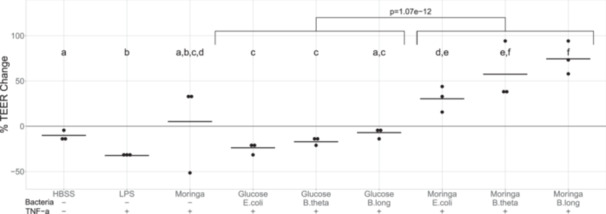
Effects of *M. oleifera* fermentations on gut barrier function. Differentiated Caco‐2 monolayers on Transwells were rinsed with HBSS and their TEER was measured before starting experimental treatments. The following were applied to Transwell inserts on their apical side: 250 µL HBSS as a mock treatment, 10 µg/mL LPS in 250 µL HBSS as a positive control to decrease TEER, 250 µL 50 mg/mL unfermented *M. oleifera* in YCFA‐NCS medium, or 250 µL bacterial fermentations of either 6 g/L glucose or 50 mg/mL *M. oleifera* leaf extract in YCFA‐NCS medium. Fresh complete DMEM medium was added to the basolateral side of Transwells at the same time as the apical treatments. After 2 h of pre‐incubation with the apical treatments, 50 ng/mL final TNF‐α was spiked into basolateral medium of all Transwells except for the mock treatments to induce barrier disruption. Twenty‐4 h after the addition of TNF‐α, posttreatment TEERs were measured from HBSS‐rinsed Transwells, and percent change in TEER calculated. Statistical significance: treatment conditions not sharing the same letter are significantly different from each other, *p* < 0.05 in pairwise comparisons. *N* = 3 independent experiments.

Mock treatment of Caco‐2 monolayers with only HBSS on the apical Transwell side caused a slight drop in TEER after 24 h (−9.0% ± 3.2%, Figure 5), and positive control treatment with 10 µg/mL lipopolysaccharide (LPS) in 250 µL HBSS (on the apical side) plus 50 ng/mL TNF‐α (on the basolateral side) decreased TEER by 30.6% ± 4.0%. Following a 2 h preincubation with fermentation supernatants on the apical side, Caco‐2 monolayers were challenged with the addition of 50 ng/mL TNF‐α to the basolateral medium. *E. coli* and *B. thetaiotaomicron* fermentations of glucose provided no protective effect against TNF‐α‐induced challenge on barrier integrity, and TEER readings decreased by 23.8% ± 5.8% and 17.1% ± 4.8%, respectively. TEER of monolayers incubated with glucose fermented by *B. longum* decreased to a lesser extent, only by 7.0% ± 6.0%, and this decrease was not significantly different than the percent change in TEER of HBSS (mock)‐treated Transwells, indicating *B. longum* fermentation negated the effect of TNF‐α.

In contrast to glucose, fermentations of *M. oleifera* leaf extract overall significantly increased Caco‐2 monolayer TEER within 24 h (*M. oleifera* vs. glucose in a two‐way ANOVA on untransformed values, *p* = 1.07e‐12,) and the magnitude of TEER increase induced by *M. oleifera* fermentations was dependent on the bacterium. TEER was increased by 30.4% ± 14.1% with *E. coli* fermentations of *M. oleifera* leaf extract. *B. thetaiotaomicron* caused greater TEER increases of 57.4% ± 33.4%, although this was not significantly different from *E. coli*. *M. oleifera* leaf extract fermented by *B. longum* increased TEER significantly more than *E. coli* (2‐way ANOVA on untransformed values followed by Tukey multiple comparisons of means, *p* = 0.002). Overall, for both glucose and *M. oleifera* fermentations, increased TEER response was significantly larger for *B. longum* than for *E. coli* (2‐way ANOVA on untransformed values followed by Tukey multiple comparisons of means, *p* = 0.0007).

Unexpectedly, sterile unfermented *M. oleifera* in YCFA‐NCS applied as an apical treatment caused a TEER decline in one experiment and yet increased TEER in two of the experiments. In a follow‐up experiment, 50 mg/mL sterile *M. oleifera* prepared in HBSS instead of YCFA‐NCS medium also caused a large decrease in Caco‐2 monolayer TEER after 24 h (data not shown). Hence, unfermented *M. oleifera* may sometimes exert a paradoxical damaging effect on Caco‐2 monolayer integrity, potentially due to presence of cytotoxic compounds which may be degraded during bacterial fermentation.

## 4 Discussion

We investigated the growth, carbohydrate utilization, and polyphenol solubilization of *M. oleifera* leaf extract during fermentation by *B. longum*, *B. thetaiotaomicron*, and *E. coli*. These three bacterial species were selected for in vitro examination because they belong to genera that were previously observed to have altered relative abundance in the gastrointestinal tract of humans or animals during a dietary *M. oleifera* intervention. Previous *M. oleifera* intervention studies utilizing 16S rRNA sequence analysis to determine the composition of the gut microbiome in both animals and humans have shown increased abundance of *Bifidobacterium*, decreased abundance of *Escherichia*, and varied responses in abundance of *Bacteroides*. Additionally, these species are commonly observed within the colon of healthy U.S. adults. Therefore, fermentation of each of these bacterial species together with *M. oleifera* leaf extract can provide insight into the microbial growth and metabolism of *M. oleifera* that might potentially occur in the human gut to affect dietary *M. oleifera*‐related health outcomes.

Both growth curve measurements and the anthrone carbohydrate assay showed that all three bacteria in this study were able to use *M. oleifera* as a carbohydrate source. However, the relative ability of *B. longum* to do so was the greatest, suggesting it was more efficient in metabolizing the complex carbohydrates from *M. oleifera* compared to the other organisms. Our results showed that *B. longum* also had the highest production of soluble phenolic compounds out of the three bacteria tested. Further, *B. longum* fermented *M. oleifera* caused the greatest increase in TEER in the Caco‐2 gut barrier model.

*E. coli* growth was reduced when SCFAs were included relative to when SCFAs were absent from the media used to ferment 50 mg/mL *M. oleifera* leaf extract. This is consistent with previous findings that SCFAs can inhibit growth of *E. coli (*Ling et al. 2025; Kadry et al. 2023) and that certain SCFAs can disrupt the membrane integrity of *E. coli* strains (Desbois and Smith 2010; Royce et al. 2013). This might further indicate that SCFAs increase *E. coli*'s susceptibility to the inhibitory effect of *M. oleifera* which has previously been reported for gram negative pathogens In the mixed microbial community of an adult colon, it is likely that SCFAs would be produced by bacteria, such as *B. longum*, during consumption of *M. oleifera*. Therefore, the combination of efficient carbohydrate utilization and SCFA production of *Bifidobacterium* in the adult colon likely contributes significantly to decreased relative abundance of organisms like *E. coli* after *M. oleifera* consumption.

*B. longum* fermentation of *M. oleifera* showed the highest increase in measurable total polyphenol content in comparison to the other two microbes tested regardless of media type. The ability of *B. longum* to increase the total polyphenolics in solution has been observed previously. The capacity for flavonoid deglycosylation has been observed in a number of *Bifidobacterium* species and while this activity was relatively low in *B. longum* isolates it was still detectable (Marotti et al. 2007; Hu et al. 2024). Additionally, a conserved locus has been identified in the *B. longum* genome for an enzyme, hydroxycinnamic acid esterase, that detaches hydroxycinnamic acid, a phenolic compound, from complex carbohydrates (Kelly et al. 2018). Chlorogenic acid, a derivative of hydroxycinnamic acid (Alam et al. 2016) has been observed in *M. oleifera* leaf extract (Amaglo et al. 2010; Bennett et al. 2003) and can be further metabolized to caffeic acid by certain strains of *Bifidobacterium* by feruloyl esterase activity (Raimondi et al. 2015). Therefore, it is reasonable to hypothesize that the increase in soluble polyphenols detected during *M. oleifera* fermentation by *B. longum* in this study could be due to the deglycosylation activity of a hydroxycinnamic acid esterase and possibly other related enzymes.

Supernatant from *B. thetaiotaomicron* fermentations of *M. oleifera* leaf extract had highly variable concentrations of measurable polyphenols. While previous studies have shown several species of *Bacteroides* have enzymatic capacity to deglycosylate rutin (quercetin‐3‐O‐glucorhamnoside) via beta glucosidase activity to quercetin (Bokkenheuser et al. 1987; Braune and Blaut 2016; Yang et al. 2012). Rutin has been identified in *M. oleifera* leaves, although the amount varied among studies (Pareek et al. 2023; Ganjayi et al. 2023; Habtemariam and Varghese 2015; Vergara‐Jimenez et al. 2017). Additionally, phenolics found in *M. oleifera* leaf extracts have been shown previously (Lee et al. 2006), as well as in this study, to moderately inhibit the growth of *B. thetaiotaomicron*. Therefore, it is possible that subtle differences in growth phase of *B. thetaiotaomicron* may have affected its susceptibility to other polyphenols present in the *M. oleifera* leaf extract.

The amount of total soluble polyphenols released during *E. coli* fermentation of *M. oleifera* was not significantly different from either of the other two microbes tested. *E. coli*, like *Bifidobacterium* also has demonstrated capacity for deglycosylation of isoflavone glycosides (Hur et al. 2000) which might have contributed to our result. The total polyphenol amount detected during *E. coli* fermentation of *M. oleifera* seemed to be impacted by the presence of both carbohydrates and SCFAs, although this visual trend was not statistically significant. The highest amount of soluble polyphenols was detected after fermentation in YCFA complete media with 50 mg/mL *of M. oleifera*, while YCFA‐NC media, lacking carbohydrates showed a slight reduction, and YCFA‐NCS showed an even greater reduction. We might predict that reduced overall metabolic activity of *E. coli* was responsible for altered polyphenol metabolism, however, we observed that change in *E. coli* total growth was greater in YCFA‐NCS than YCFA‐NC, which is counter to this hypothesis. Therefore, further studies are needed to understand this activity of *E. coli*.

In general, the fermentation of *M. oleifera* leaf extract led to increased TEER in a Caco‐2 gut barrier model. Tight junctions can be modulated by plant phenolics. Quercetin, kaempferol, procyannidins, and ferulic acids have all been shown to increase TEER (Kosińska and Andlauer 2013). In *M. oleifera* leaf extracts, glycosidic forms of quercetin (i.e., quercetin 3‐O‐galactoside, quercetin 3‐O‐glucoside, and quercetin 4'‐O‐glucoside) were the most represented compounds among flavonoids (Rocchetti et al. 2019). It is therefore possible that in addition to metabolism of hydroxycinnamic acid derivatives, one of the ways in which bacterial fermentation of *M. oleifera* leads to increased TEER, is through metabolism of quercetin glycosides to increase soluble quercetin. Interestingly, unfermented *M. oleifera* leaf extract led to mixed effects on TEER. Methanol and dichloromethane *M. oleifera* leaf extracts have been shown to be cytotoxic to Caco‐2 cells in ~250−100 μg/mL doses (Suphachai 2014). It is possible that cytotoxic compounds such as arsenic compounds which are sometimes absorbed by *M. oleifera* from soil, were variably extracted with water, but the cytotoxic effect was negligible relative to beneficial compounds, including SCFAs, generated during fermentation.

In conclusion, of the three bacteria tested, *B. longum* was able to grow most efficiently using *M. oleifera* as a carbohydrate source and produced the highest amount of soluble polyphenols during fermentation. Additionally, *B. longum*‐fermented *M. oleifera* significantly increased TEER readings in a Caco‐2 monolayer, a surrogate measurement for gut barrier function. It is therefore possible that *B. longum* fermentation of *M. oleifera* during human consumption could help facilitate the reduction in relative abundance of gram‐negative microorganisms like *E. coli* and may mediate some of the health benefits received from production of SCFAs and soluble polyphenols.

## Author Contributions

**Mary E. Kable:** conceptualization (equal), formal analysis (lead), supervision, writing – original draft preparation (lead), writing – review and editing. **Carrie Waterman:** conceptualization (equal), writing – review and editing (equal). **Danielle G. Lemay:** conceptualization (equal), writing – review and editing (equal). **David H. Storms:** investigation (lead), methodology (equal), writing – original draft preparation (supporting). **Zeynep Alkan:** investigation (equal), methodology (supporting), writing – original draft preparation (supporting). **Dana DeVries:** investigation (equal), methodology (equal), writing – original draft preparation (supporting). **Maneesha Muriki:** investigation (suppporting), writing – original draft preparation (supporting). **Carrie Waterman:** was a faculty member at UC Davis at the time of research, and currently at the CA Dept of Food & Agriculture (carrie. waterman@cdfa. ca. gov).

## Ethics Statement

The authors have nothing to report.

## Consent

All authors have approved the publication of this manuscript.

## Conflicts of Interest

The authors declare no conflicts of interest.

## Supporting information


**Supplemental Figure 1:** *M. oleifera* leaf extract effects on total growth of bacterial isolates in complete media. **Supplemental Figure 2:** *M. oleifera* leaf extract effects on total growth of bacterial isolates in minimal media.
