# Supplementary material for: Gut Microbe Fermentation of Moringa oleifera Leaf Extract Increases Measurable Polyphenols and Improves Barrier Function in a Cell Culture Model
Source: Microbiologyopen. 2025 Nov 24;14(6):e70068. doi: 10.1002/mbo3.70068 (PMC12643947; doi:10.1002/mbo3.70068)
Supplement: Supplementary file 1 — Supplemental Figure 1: M. oleifera leaf extract effects on total growth of bacterial isolates in complete media. Supplemental Figure 2: M. oleifera leaf extract effects on total growth of bacterial isolates in minimal media. [file MBO3-14-e70068-s001.docx]

**Supplemental Figure 1. *M. oleifera* leaf extract effects on total growth of bacterial isolates in complete media.** Sum growth measured by net increase in A600 of bacterial isolates in complete YCFA media containing moringa leaf extract concentrations shown on the x-axis. Results of repeated measures ANOVA (*P* shown on each graph) with Dunnett’s post-test using total growth at 0 mg/mL as a reference to compare to total growth at each extract concentration with Log_10_ (*B. longum*, *E. coli*) or Tukey’s Ladder of Powers (*B. thetaiotamicron*) transformed data. Bonferroni correction was performed for multiple comparisons. Statistical significance for three independent experiments: p < 0.05 *, p < 0.01 **, p < 0.001 ***

**Supplemental Figure 2. *M. oleifera* leaf extract effects on total growth of bacterial isolates in minimal media.** Sum growth measured by net increase in A600 of bacterial isolates in YCFA-NCS media, lacking carbohydrates and SCFAs, containing moringa leaf extract concentrations shown on the x-axis. Results of repeated measures ANOVA (*P* shown on each graph) with Dunnett’s post-test using total growth at 0 mg/mL as a reference to compare to total growth at each extract concentration with raw data (*B. longum*), and Tukey’s Ladder of Powers (*B. thetaiotamicron*) or cube root (*E. coli*) transformed data. Bonferroni correction was performed for multiple comparisons. Statistical significance for three independent experiments: p < 0.05 *, p < 0.01 **, p < 0.001 ***
